# Biological characteristics associated with virulence in *Clostridioides difficile* ribotype 002 in Hong Kong

**DOI:** 10.1080/22221751.2020.1739564

**Published:** 2020-03-17

**Authors:** Ka Yi Kong, Thomas N. Y. Kwong, Hung Chan, Kristine Wong, Samuel S. Y. Wong, Anu P. Chaparala, Raphael C. Y. Chan, Lin Zhang, Joseph J. Y. Sung, Jun Yu, Peter M. Hawkey, Margaret Ip, William K. K. Wu, Sunny H. Wong

**Affiliations:** a Institute of Digestive Disease, Department of Medicine and Therapeutics, The Chinese University of Hong Kong, Hong Kong, Hong Kong SAR; b State Key Laboratory of Digestive Disease, Li Ka Shing Institute of Health Sciences, Faculty of Medicine, The Chinese University of Hong Kong, Hong Kong, Hong Kong SAR; c Department of Anaesthesia and Intensive Care, Faculty of Medicine, The Chinese University of Hong Kong, Hong Kong, Hong Kong SAR; d Division of Biological Sciences, University of California San Diego, San Diego, CA, USA; e Department of Microbiology, Faculty of Medicine, The Chinese University of Hong Kong, Hong Kong SAR; f CUHK Shenzhen Research Institute, The Chinese University of Hong Kong, Shenzhen, People’s Republic of China; g Institute of Microbiology and Infection, School of Biosciences, University of Birmingham, Birmingham, UK; h Department of Microbiology, Faculty of Medicine, The Chinese University of Hong Kong, Hong Kong, Hong Kong SAR

**Keywords:** *Clostridioides difficile*, ribotype, sporulation, germination, toxins

## Abstract

*Clostridioides difficile* infection (CDI) is a common cause of nosocomial diarrhea and can sometimes lead to pseudo-membranous colitis and toxic megacolon. We previously reported that the PCR ribotype 002 was a common *C. difficile* ribotype in Hong Kong that was associated with increased mortality. In this study, we assessed *in vitro* bacteriological characteristics and *in vivo* virulence of ribotype 002 compared to other common ribotypes, including ribotypes 012, 014 and 046. We observed significantly higher toxin A (*p <* 0.05) and toxin B (*p *< 0.05) production, sporulation (*p <* 0.001) and germination rates (*p *< 0.0001) in ribotype 002 than other common ribotypes. In a murine model of *C. difficile* infection, ribotype 002 caused significantly more weight loss (*p* < 0.001) and histological damage (*p* < 0.001) than other common ribotypes. These findings may have contributed to the higher prevalence and mortality observed, and provided mechanistic insights that can help public surveillance and develop novel therapeutics to combat against this infection.

## Introduction

*Clostridioides difficile* infection is caused by an anaerobic, spore-forming Gram-positive bacterium, *C. difficile* [1]. It is the most common cause of nosocomial gastrointestinal infection and represents a formidable public health challenge [2]. In the United States (US), it was estimated that there were 453,000 incidence cases and around 29,000 related deaths in 2011 [3]. This bacterium can colonize the human gastrointestinal tract and proliferate to cause disease especially after antibiotic treatments that change the colonic microbiota and reduce colonization resistance [1]. Different bacteriological mechanisms have been studied, including toxin production [4], spore formation and germination [5,6]. Notwithstanding, it was suggested that the bacteriological and pathogenic characteristics of different isolates of *C. difficile* were not thoroughly studied in Asia [7].

*C. difficile* can secrete two large clostridial toxins, toxin A (TcdA) and toxin B (TcdB) [5,6], as well as a binary toxin (CDT; also known as *C. difficile* transferase) that only exists in certain ribotypes such as ribotype 027 [1,8]. Toxin A and toxin B are responsible for causing inflammation and necrosis of the intestinal epithelial cells [5,6]. CDT is made up of two components: the enzymatic component that performs actin modification through ADP-ribosyltransferase activity and the binding component that anchor to the host cells [8]. In a previous study, toxigenic strains were able to produce increased levels of toxins A and B which attained highest concentrations at 72 h [9].

Under unfavourable conditions, such as lack of nutrients and other stress factors, *C. difficile* can survive by producing dormant spores and transmit within the hospital environment [10–16]. These spores are resistant to environmental factors including heat, acid and antibiotics [10–15]. Once conditions become favourable again, these aerotolerant spores can germinate into vegetative cells [17]. Ribotype 014/020 was also found to have slightly elevated sporulating level at 24 h but the more prevalent ribotype 002 was able to sporulate at a later time point at 72 h [18]. In another study, the percentage of aerotolerant spores in ribotype 002 was 20.2% which was significantly higher than that of other 56 strains of different ribotypes [19].

Importantly, some of these virulence mechanisms are accountable for the clinical outcomes of CDI. For instance, higher levels of toxins [20] and sporulation rates [21] have been reported for *C. difficile* BI/NAP1/027, a hypervirulent ribotype that has caused epidemic outbreaks and is prevalent across developed countries such as North America and Western Europe [22–29]. This strain can also produce the binary toxin [1], which was suggested to potentiate the toxicity of TcdA and TcdB to increase the severity of diseases [8,30,31]. Meanwhile, different bacterial ribotypes have been reported in studies from different countries. For example, 98 ribotypes were found among 720 toxigenic isolates collected in six centres in the US [28,32], 32 ribotypes existed in 70 samples in a study from Australia [33], and 86 ribotypes were identified from 705 *C. difficile* isolates in five centres in West London in the United Kingdom [34]. These suggested that the epidemiology of *C. difficile* was not only caused by one or a few hypervirulent ribotypes but also included a diversity of strains in causing the infection. In Asia, hypervirulent ribotype 027 strain only occurred sporadically, with the most prevalent ribotypes being 002, 017, 018, 014 and 012 [19,35–41]. In Hong Kong, the rising incidence and disease burden of CDI have been reported, with the most common ribotypes being 002 (22.8%), 012 (14.1%), 014 (14.1%) and 046 (13.0%) [42]. Notably, ribotype 002 was associated with increased mortality at 47.6% at 30 days after CDI diagnosis, compared to 12.7% for other ribotypes [42]. These data translated into an adjusted hazard ratio of 2.8 [42] for death in patients infected with *C. difficile* ribotype 002. In fact, ribotype 002 was also one of the most prevalent ribotypes circulating in Australian hospitals in recent years [43].

Based on these clinical observations, we hypothesized that *C. difficile* ribotype 002 may have distinct microbiological features that promotes its virulence capacity in comparison to other common ribotypes.

## Materials and methods

### Experimental groups

*C. difficile* isolates used in this project were collected at the Prince of Wales Hospital in Hong Kong between 2009 and 2011 [44]. A total of 20 strains of five ribotypes were chosen for subsequent experiments.

We performed bacteriological characteristics and murine model experiments on *C. difficile* ribotype 002 and other common ribotypes including 012, 014 and 046, which together, constituted to 64% of all CDI incidences in Hong Kong [42]. *C. difficile* ribotypes 027 and 087 were also included as positive controls. The groups used in the experiments are defined as below: Group A (ribotype 002), Group B (ribotypes 012, 014, 046), Group C (ribotype 027), Group D (ribotype 087 (VPI 10463)) and Group E (Brain Heart Infusion (BHI) control). Five separate isolates per ribotype 002 and 012, and four isolates per ribotype 014 and 046 were chosen. Only two local strains of ribotype 027 were available and were included in this study.

### C. difficile culture conditions and growth

*C. difficile* strains were cultured in BHI at 37°C in an anaerobic chamber (BACTRON 300, Sheldon Manufacturing) maintained in a gas mixture of 5% H_2_, 5% CO_2_, and 90% N_2_ [45,46]. All growth medium and solutions were pre-reduced in the anaerobic chamber one day prior usage [45,46]. The overnight grown cultures were adjusted to optical density 600 nm (OD_600_) of 0.05 with fresh pre-reduced medium and recorded as 0 h, and subsequently measured at specific time points, i.e. 0, 4, 8, 12, 16, 20, 24, 32 and 48 h with a spectrophotometer (NanoDrop 2000c spectrophotometer, Thermo Scientific). Briefly, 1 ml aliquot of culture broth was collected at designated time points and spread onto BHI agars for enumeration of bacterial counts in colony-forming units (CFUs) after 24 h incubation anaerobically.

### Toxin production

Toxin production from different bacterial strains were measured by an enzyme-linked immunosorbent assay (ELISA) (Separate detection of *C. difficile* toxins A and B kit; TGC-E002-1; tgcBiomics) according to manufacturer’s instruction. Supernatants from *C. difficile* culture were collected by centrifugation at 2500 × *g* for 5 min and filtered through 0.22 μm membrane at 24, 48, and 72 h, respectively. Bacterial supernatants were diluted accordingly before placing in the wells of ELISA plates for toxin detection. Measurements were performed in duplicates.

### Sporulation and germination

Only *C. difficile* spores, but not vegetative cells, can survive heat above physiological temperature. To recover colonies on agar plates [47], *C. difficile* spores were allowed to germinate in the presence of sodium taurocholate [48]. Aliquots of overnight cultures were resuspended in fresh medium and adjusted to OD_600_ 0.1 and incubated anaerobically to OD_600_ 0.2–0.5 [49]. This time point was recorded as 0 h and two tubes of culture were collected [49]. One tube was heated at 70°C for 25 min to kill the vegetative cells, remained the spores. Another tube was not heated and contained both the vegetative cells and spores. The samples were spread on BHI agars with or without taurocholate [49] and bacterial CFU counts were enumerated after overnight incubation. Sporulation frequencies were calculated as CFUs of heated samples divided by those of unheated samples plated on BHI agars with 0.1% sodium taurocholate. Germination was used to assess the ability of spores to outgrow in the absence of taurocholate [50], and were calculated as CFUs of heated samples on agars without sodium taurocholate divided by those on agars with sodium taurocholate. Each strain was serially diluted, and four dilutions were spread on the plates. CFU of each ribotype was calculated by averaging the CFUs of the strains in that ribotype. Collection of samples and spreading on agars were repeated for every 24 h, the last time point was 120 h.

### Mouse model

C57BL/6 male mice of 3–4 weeks old were used in this study. Cefoperazone (0.5 mg/ml) were supplemented in their drinking water for 10 days to disrupt intrinsic colonization resistance by commensal microbiota, render the mice susceptible to *C. difficile* infection [51]. During the 10-day antibiotics treatment, the drinking water was changed every other day to prevent the breakdown of antibiotics and was switched to normal water for two days prior to oral gavage [52]. After 12 days of pre-treatment, the mice were divided into groups and orally gavaged with 10^8^ CFU of vegetative *C. difficile* cells of different ribotypes. The grouping was the same as the part of bacteriological characteristics, while a group of positive control gavaged with a *C. difficile* standard strain (VPI 10463 of ribotype 087) and a group of negative control gavaged with BHI medium were added in animal model. The day of gavage was recorded as day 0 and the mice were sacrificed on day 2 [52].

The animal study was performed at the Laboratory Animal Services Centre in compliance with the institutional requirements. Animal ethics approval (AEEC approval number: 16/056/GRF-4-B) was obtained from the Animal Experimentation Ethics Committee (AEEC) of the Chinese University of Hong Kong.

### Mice overall condition and body weight

The overall condition, including posture, motility, eye and hair conditions, body weight and survival of mice were monitored after bacterial inoculation. Body weight of each mouse was measured daily from day 0 to day 2. Body weights on day 0 were regarded as 100% as the baseline weight for each mouse, the data shown as percent baseline weight as compared to day 0.

### Histological assessment

The mice were sacrificed on day 2. Colon samples were placed in 10% buffered formalin for 24 h and then stored in 70% ethanol [52]. The samples were further processed and embedded in paraffin before sectioning [52]. After sectioning at a thickness of 4 µm, the slides were stained with haematoxylin and eosin (H&E) and observed under 100 × magnification (Olympus DP80) [52]. Histological scores were graded according to a histology scoring system [51], based on three criteria of edema, cellular infiltration and epithelial changes (refer to Supplementary Table 1).

### Data analysis

The data of growth, toxin concentrations, sporulation and germination and mice body weight were calculated using two-way analysis of variance (ANOVA) with Tukey’s post hoc testing using GraphPAD PRISM (v8.0.1). Error bars were plotted with mean ± estimated standard error. The threshold of *p* < 0.05 was used to indicate statistical significance. Data from the histology scores in animal model part were analyzed with one-way ANOVA with Tukey’s post hoc testing by GraphPAD PRISM.

## Results

### Ribotype 002 showed extended stationary phase

**Figure 1.**
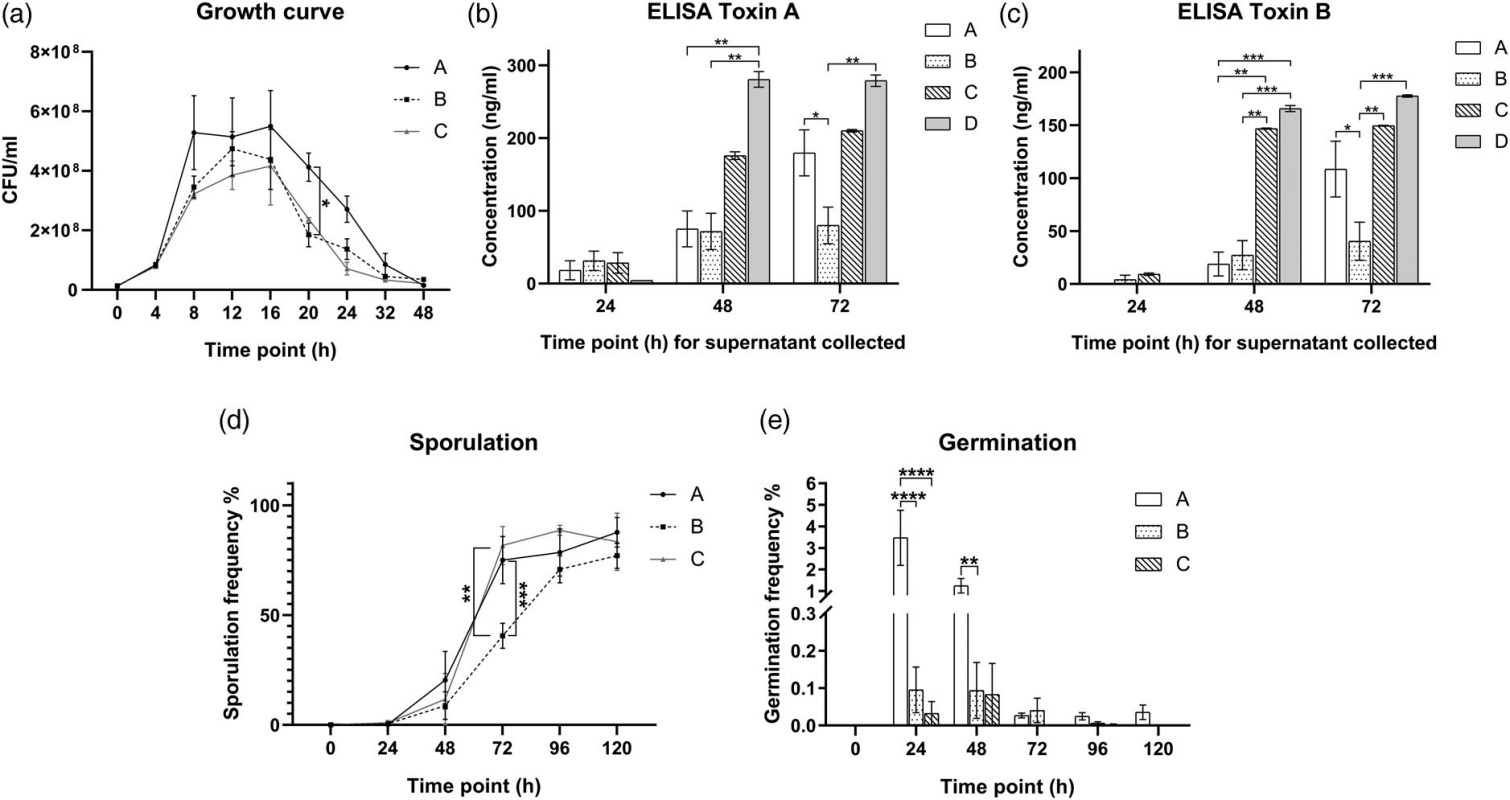
(A) Bacteriological characteristics: Growth curves of various *C. difficile* strains at 9 time points. (B) Bacteriological characteristics: Production of toxin A, by different *C. difficile* strains at 3 time points as determined by ELISA. (C) Bacteriological characteristics: Production of toxin B, by different *C. difficile* strains at 3 time points as determined by ELISA. (D) Bacteriological characteristics: Sporulation frequency (%) by different *C. difficile* strains at 6 time points. (E) Bacteriological characteristics: Germination frequency (%) of different *C. difficile* strains at 6 time points. Group A, ribotype 002 (*n* = 5); Group B, other ribotypes including ribotypes 012, 014 and 046 (*n* = 13); Group C, ribotype 027 (*n* = 2); Group D, ribotype 087 (*n* = 7) of standard strain (VPI10463). *p < 0.05; **p < 0.01; ***p < 0.001; ****p < 0.0001 significantly different between the indicated groups.

We studied the *in vitro* dynamics of bacterial growth in culture. Despite similar growth at the lag phase at 4 h, higher CFUs were reached for *C. difficile* ribotype 002 (Group A) at stationary phase (Figure 1(A)). During the exponential phase (4–8 h), the average generation time for ribotype 002 was 100.09 min (SD = 23.5 min), which was shorter than other common ribotypes of 123.22 min (SD = 26.2 min). Remarkably, ribotype 002 had higher vegetative cell numbers than other common ribotypes, with a significant statistical difference observed at 20 h (Group A versus Group B: 4.13 × 10^8^ CFU/ml versus 1.85 × 10^8^ CFU/ml, *p *< 0.05, Figure 1(A)).

### Ribotype 002 produced higher levels of toxins A and B

Toxin A and B concentrations for the various ribotypes are shown in Figure 1(B,C) respectively. Ribotype 002 was compared to other ribotypes, including ribotype 027 and the standard strain VPI 10463, which are known to be hyper-toxin-producing. All *C. difficile* non-027 ribotypes except VPI 10463 produced low levels of toxins at 48 h (Figure 1(B,C)). Nevertheless, at 72 h, ribotype 002 produced significantly higher levels of toxin A (Group A versus Group B: 179.7 ng/ml versus 80.07 ng/ml, *p <* 0.05, Figure 1(B)) and toxin B (Group A versus Group B: 108.5 ng/ml versus 40.44 ng/ml, *p *< 0.05, Figure 1(C)) when compared to other common ribotypes. Indeed, there were no significant differences between the toxin levels of ribotypes 002 and 027 at 72 h. The toxin levels of different ribotypes, including that of ribotype 027 and test strain VPI 10463, were illustrated in Figure 1(B,C).

### Ribotype 002 exhibited higher sporulation and germination rates

Low sporulation rates were observed across ribotypes at 0 and 24 h, likely due to the presence of sufficient nutrients and favourable environment in the culture media as shown in Figure 1(D). This slow rate of sporulation extended into the early stationary phase. Sporulation increased during the time from 48 to 120 h, especially with ribotypes 002 and 027, which showed significantly higher sporulation rates than other common ribotypes at 72 h (Group A versus Group B: 75.05% versus 40.57%, *p <* 0.001; Group C versus Group B: 81.68% versus 40.57%, *p *< 0.01; Group A versus Group C: 75.05% versus 81.68%, *p* > 0.5, Figure 1(D)). The sporulation rates of other ribotypes increased at 96 and 120 h, with no significant differences among the groups at these later time points.

We then measured the germination rates of different *C. difficile* ribotypes with addition of sodium taurocholate. We observed increased germination rates of ribotype 002 compared to other ribotypes at 24 h (Group A versus Group B: 3.472% versus 0.113%, *p *< 0.0001; Group A versus Group C: 3.472% versus 0.032%, *p *< 0.0001, Figure 1(E)) and 48 h (Group A versus Group B: 1.245% versus 0.094%, *p *< 0.01; Group A versus Group C: 1.245% versus 0.083%, *p* > 0.05, Figure 1(E)). This indicates that a higher proportion of spores in ribotype 002 can germinate spontaneously in the absence of sodium taurocholate than other common ribotypes. The germination frequencies dropped in the subsequent time points.

### Ribotype 002 induced more weight loss and caused more severe histological damage in a murine CDI model

**Figure 2:**
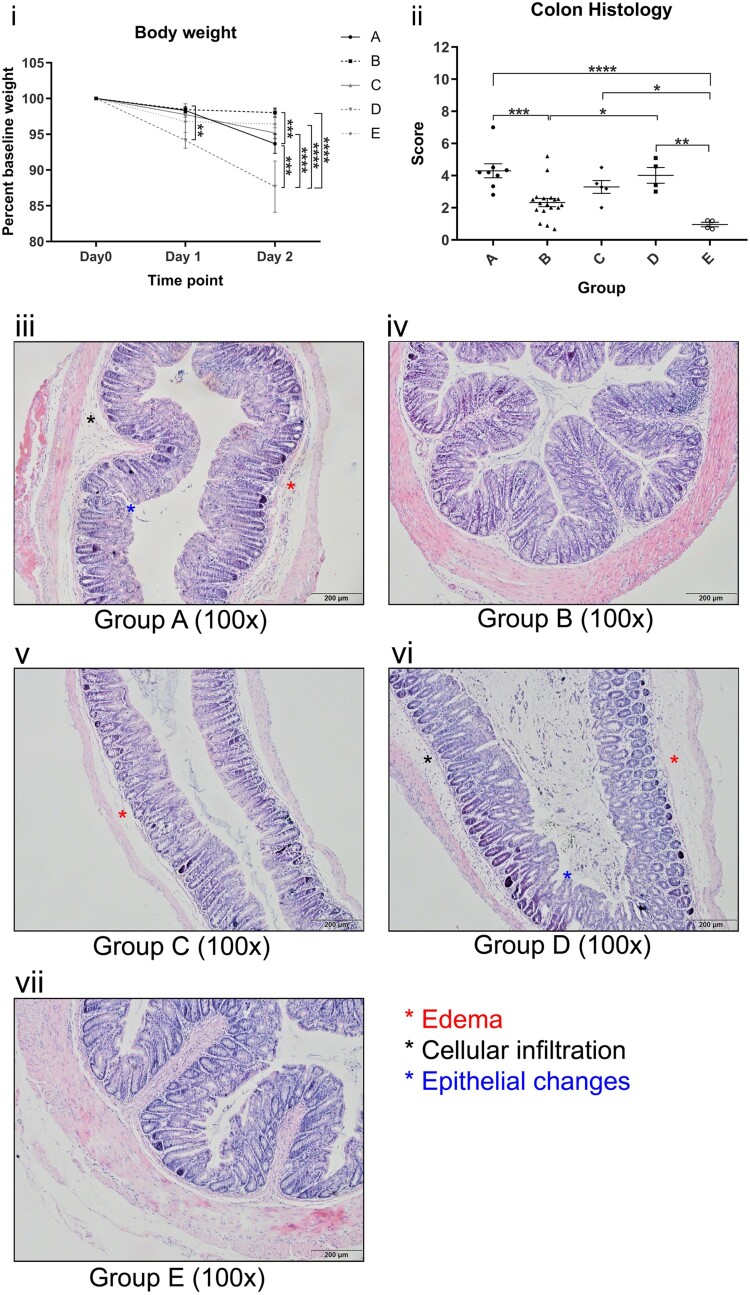
. (i) Body weight of mice gavaged with different *C. difficile* ribotypes. (ii) Total histology scores of H&E stained colon tissues of mice at the time of sacrifice (day 2) with different *C. difficile* ribotypes. Group A, ribotype 002 (*n* = 8); Group B, other ribotypes, include ribotypes 012, 014, and 046 (*n* = 21); Group C, ribotype 027 (*n* = 8); Group D, ribotype 087 (*n* = 7) of standard strain (VPI10463); Group E, BHI control group (*n* = 4). *p < 0.05; **p < 0.01; ***p < 0.001; ****p < 0.0001 significantly different between the indicated groups. (iii–vii) Histology photos of H&E stained colon tissues from each group of different ribotypes; Group B and E demonstrate normal colon sections with no significant edema, cellular infiltration and epithelial changes; Group A, C and D show some signs of significant edema, cellular infiltration and epithelial changes when compared to Group B and E.

All groups experienced body weight drop, with a mean weight loss of 2.9% on day 1 and 5.8% on day 2 after oral gavage of *C. difficile* (Figure 2(i)). At day 2, ribotype 087 experienced the most significant body weight drop of 12.32%, followed by ribotype 002 with 6.32% weight loss and was statistically significant when compared to other common ribotypes (Group A versus Group B: 6.32% versus 1.97%, *p *< 0.001, Figure 2(i)). The remaining three groups did not show significant differences in weight from day 0 to day 2.

On day 2, three out of eight mice gavaged with ribotype 027 (37.5%) and two out of seven mice gavaged with ribotype 087 (28.6%) were found to be dead. Dead mice were excluded for collecting histological samples. Although all mice survived in other groups, one mouse in ribotype 002 showed signs of distress, as indicated by a hunched posture, sluggish movement, and rough hair coat. No obvious appearances or behavioural abnormalities were observed in mice from other groups.

The intestinal inflammation and injury in the colon of the mice as induced by *C. difficile* were graded according to criteria as defined by a histological score (Supplementary Table 1). There was no statistical difference in the histology score of ribotypes 002, 027 and 087 (Group A versus Group C: 4.296 versus 3.294, *p* > 0.1; Group A versus Group D: 4.296 versus 4.016, *p* > 0.5). Notably, ribotype 002 had significantly higher scores than other common ribotypes (4.296 versus 2.324, *p *< 0.001) and BHI control (4.296 versus 0.9511, *p *< 0.0001), indicating severe inflammation and tissue injury in the colon. Representative H&E histological sections of the colon from the groups are shown in Figure 2(iii–vii), revealing significant submucosal edema, neutrophilic infiltration and epithelial cell damage with ribotypes 002, 027 and 087. The respective mean histological scores of each group of mice are shown in Figure 2(ii).

## Discussion

BI/NAP1/027, the hypervirulent strain that related to epidemic CDI outbreaks in the US and Europe, only occurred sporadically in Hong Kong. Ribotype 002 is the most common ribotype in Hong Kong, followed by ribotypes 012, 014 and 046 [42]. Herein, the bacteriological characteristics of ribotype 002 and *in vivo* virulence was further investigated in comparison to ribotypes 012, 014 and 046.

Ribotype 002 showed extended stationary phase, heightened sporulation and germination frequencies, and higher toxin production than other common ribotypes in Hong Kong. It was suggested that the rate of sporulation was associated with the severity of CDI outbreak [53], and enhanced sporulation capacity was also suggested to be associated with greater spread or persistence in hospital settings [54,55]. Ribotype 002 had a comparable high sporulation frequency to produce spores as ribotype 027; ribotypes 002 and 027 showed similar rising trend along the time points. The hypervirulent ribotype 027 strain was reported to sporulate earlier and accumulate with more spores than non-hypervirulent strains [56]. Here, we demonstrated similar pattern of earlier onset and higher sporulation frequencies for both ribotypes 002 and 027. This may indicate that prevalence of ribotype 002 in Hong Kong is related to its virulence in terms of sporulation. A previous study suggested that the concentration of taurocholate was trivial in the lower intestine as it was deconjugated [57]. Meanwhile, ribotype 002 spores had a better ability to germinate without taurocholate when compared to all other ribotypes, including ribotype 027. Thus, it may indicate that the higher germination frequency of ribotype 002 in the absense of taurocholate may be associated with the increased rates of reinfection of CDI [58].

Compared to other common ribotypes, ribotype 002 showed a few clinical complications on mice, such as body weight lost and high histology scores; yet, no mice were found dead. However, more severe disease severity was observed on the VPI 10463 (ribotype 087) and ribotype 027 with reduced percentage survival.

The virulence characteristics of ribotype 002 may relate to its high prevalence and higher mortality in Hong Kong. Earlier onset of sporulation of ribotype 002 than other common ribotypes may also account for its persistence in the environment. The higher spontaneous germination rate of ribotype 002 may imply that its spores are capable to germinate even under not very favourable conditions. Although clinical features on mice gavaged with *C. difficile* ribotype 002 were not as severe as those with hypervirulent ribotype 027 and high-toxin-producer standard strain VPI 10463, obvious differences existed in terms of body weight lost and higher histology scores when compared to other common ribotypes. This demonstrated that ribotype 002 had a higher ability to cause disease with clinical complications on animals than other common ribotypes. Our study demonstrated the fitness and a potentially important strain from transmission and severity that deserve further attention and surveillance.

## Supplementary Material

Supplemental Material
